# Virus Enhanced Microrobots for Biofilm Eradication

**DOI:** 10.1002/adma.202508299

**Published:** 2025-10-04

**Authors:** Jyoti, Sagar Arya, Xia Peng, Martin Pumera

**Affiliations:** ^1^ Future Energy and Innovation Laboratory Central European Institute of Technology Brno University of Technology (CEITEC‐BUT) Brno 61200 Czech Republic; ^2^ Faculty of Electrical Engineering and Computer Science VSB–Technical University of Ostrava 17. listopadu 2172/15 Ostrava 70800 Czech Republic; ^3^ Department of Chemical and Biomolecular Engineering Yonsei University 50 Yonsei‐ro, Seodaemun‐gu Seoul 03722 South Korea; ^4^ Department of Medical Research China Medical University Hospital China Medical University No. 91 Hsueh‐Shih Road Taichung 4040 Taiwan

**Keywords:** biomedicine, infection, nanorobots

## Abstract

Biofilms pose significant challenges in biomedical, industrial, and environmental applications due to their inherent resistance to antimicrobial agents. This study introduced an innovative and effective strategy for biofilm eradication by employing virus‐conjugated microrobots (virus@microbots). These biofunctionalized microrobots are synthesized via the hydrothermal method, followed by the functionalization of the viruses. The synergy of microrobots’ active movement and the virus's specificity boosted biofilm removal by enabling targeted binding, penetration, and effective delivery into the biofilm matrices. Additionally, compared to microrobots alone, virus@microbots significantly accelerated and refined the process. By integrating biological specificity with magnetic responsiveness, this approach demonstrated the efficacy of virus@microbots as a viable antibacterial strategy for biofilm elimination, offering a promising antibacterial platform for combating biofilm‐associated infections. Future research should focus on optimizing microrobot design and viral conjugation protocols to enhance scalability and therapeutic specificity, paving the way for clinical translation and broader applications in infection control.

## Graphical abstract

Biofilms resist antimicrobials across medicine, industry, and environments. We present virus‐conjugated microrobots synthesized hydrothermally and magnetically actuated. Their motion and viral specificity enable targeted binding, penetration, and delivery, outperforming bare microrobots. This platform achieves rapid, efficient biofilm eradication; future work will optimize design and conjugation for scalable, clinically translatable infection control.

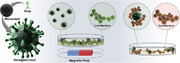

## 1 Introduction

Biofilms are communities of microorganisms that stick to different surfaces, such as medical devices, food processing equipment, industrial piping, and living tissues through the secretion of extracellular polymeric substances (EPS). These EPS matrices, primarily made of polysaccharides, proteins, and extracellular DNA, not only help the microbes to stay attached but also make them much more resistant to antibiotics and environmental stress.^[^ ^1^, ^2^ ^]^ These biofilms are highly resistant to the normal antimicrobial agents and so are hazardous for the food safety, healthcare, and environmental settings,^[^ ^3^, ^4^ ^]^ because of this, they pose serious risks to healthcare, industry, and the environment. It's estimated that over 60% of microbial infections in humans are associated with biofilms, often contributing to long‐lasting and difficult‐to‐treat diseases.^[^ ^5^, ^6^, ^7^ ^]^

One of the most concerning biofilm‐forming bacteria is *Staphylococcus aureus*, known for its strong biofilm formation and potent toxins, contributing to severe infections ranging from minor skin issues to life‐threatening conditions like sepsis and endocarditis.

It has been reported that *S. aureus* enterotoxins were responsible for 6.4% of all reported outbreaks in the European Union in 2012, as documented by the European Food Safety Authority (EFSA) and the European Centre for Disease Prevention and Control (ECDC, 2016).^[^ ^8^, ^9^, ^10^ ^]^ In addition, these biofilms also disrupt industrial operations by leading to corrosion, contamination, and major economic loss.^[^ ^11^, ^12^, ^13^ ^]^

Many physical and chemical approaches have been explored to remove biofilms, like ultrasound, coatings, and disinfectants.^[^ ^14^, ^15^, ^16^ ^]^ One such example is the use of ultrasonic waves to remove *Salmonella enteritidis* biofilms, as reported by Zhang et al.^[^ ^17^ ^]^ Similarly, Vladkova et al. demonstrated that protecting surfaces with various antibiofilm coatings can mitigate intrusive bacterial colonization, thereby regulating bacterial attachment and subsequent biofilm formation.^[^ ^18^ ^]^ However, they often fall short due to limited efficiency, high cost, labor issues, issues with residual remains, or toxicity concerns.^[^ ^16^, ^19^, ^20^ ^]^

More recently, microrobots have emerged as a attractive alternative tool. These tiny machines can actively move through complex environments, applying force to disrupt biofilm structures. However, these microrobotic therapies frequently use antibacterial coatings, which raised concerns about cytotoxicity, and hence limit their use in biological settings.,^[^ ^21^, ^22^, ^23^, ^24^, ^25^, ^26^, ^27^, ^28^ ^]^ In contrast, virus‐based therapies have garnered attention for their ability to selectively target and eliminate biofilms without inducing antibiotic resistance.^[^ ^29^, ^30^, ^31^, ^32^, ^33^ ^]^ Bacteriophages, or simply phages, are viruses that specifically infect and kill bacteria. Due to their precision and natural origin, they've gained interest as an environmentally friendly alternative to antibiotics.^[^ ^34^, ^35^ ^]^ However, these treatments face challenges; first, viruses tend to dilute in bulk media, prolonging the duration of their active effect,^[^ ^36^, ^37^, ^38^, ^39^ ^]^ and second, their penetration into dense structures of mature biofilms remains limited.^[^ ^40^, ^41^, ^42^, ^43^ ^]^

To address this, we combine the strengths of both systems i.e., the targeting ability of viruses with the guided mobility of magnetic microrobots. By conjugating the viruses onto the surface of the magnetically actuated Fe_3_O_4_ microrobots, it formed virus@microbots. These *dual‐functional* platforms are capable of targeted navigation, mechanical disruption, and localized bactericidal activity.^[^ ^44^, ^45^, ^46^ ^]^ Prior studies have already shown that magnetic microrobots significantly improve biofilm penetration and cargo delivery, especially under external magnetic control.^[^ ^45^, ^47^, ^48^, ^49^, ^50^ ^]^

**Scheme 1:**
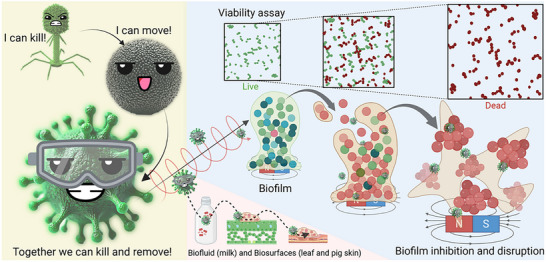
*Dual‐Action* Biofilm Removal by Virus@microbots: Illustration of the synergistic biofilm eradication by virus@microbots. Viruses deliver precise targeting and bactericidal action, while microbots provide magnetic navigation and mechanical disruption. Combined, they effectively suppress bacterial proliferation and eliminate biofilms on diverse surfaces, including milk, leaves, and chicken or porcine skin.

In this work, we develop and test virus@microbots for targeted biofilm removal. The Fe_3_O_4_ microrobots were prepared via a hydrothermal method, functionalized with viruses, and evaluated for their ability to move through and disrupt *S. aureus* biofilms. Our findings demonstrate enhanced eradication through synergistic mechanical and biological action, supporting virus@microbots as a scalable, biocompatible, and selective platform for advanced antimicrobial applications.(**Scheme** **1**)

## 2 Results and Discussion

**Figure 1:**
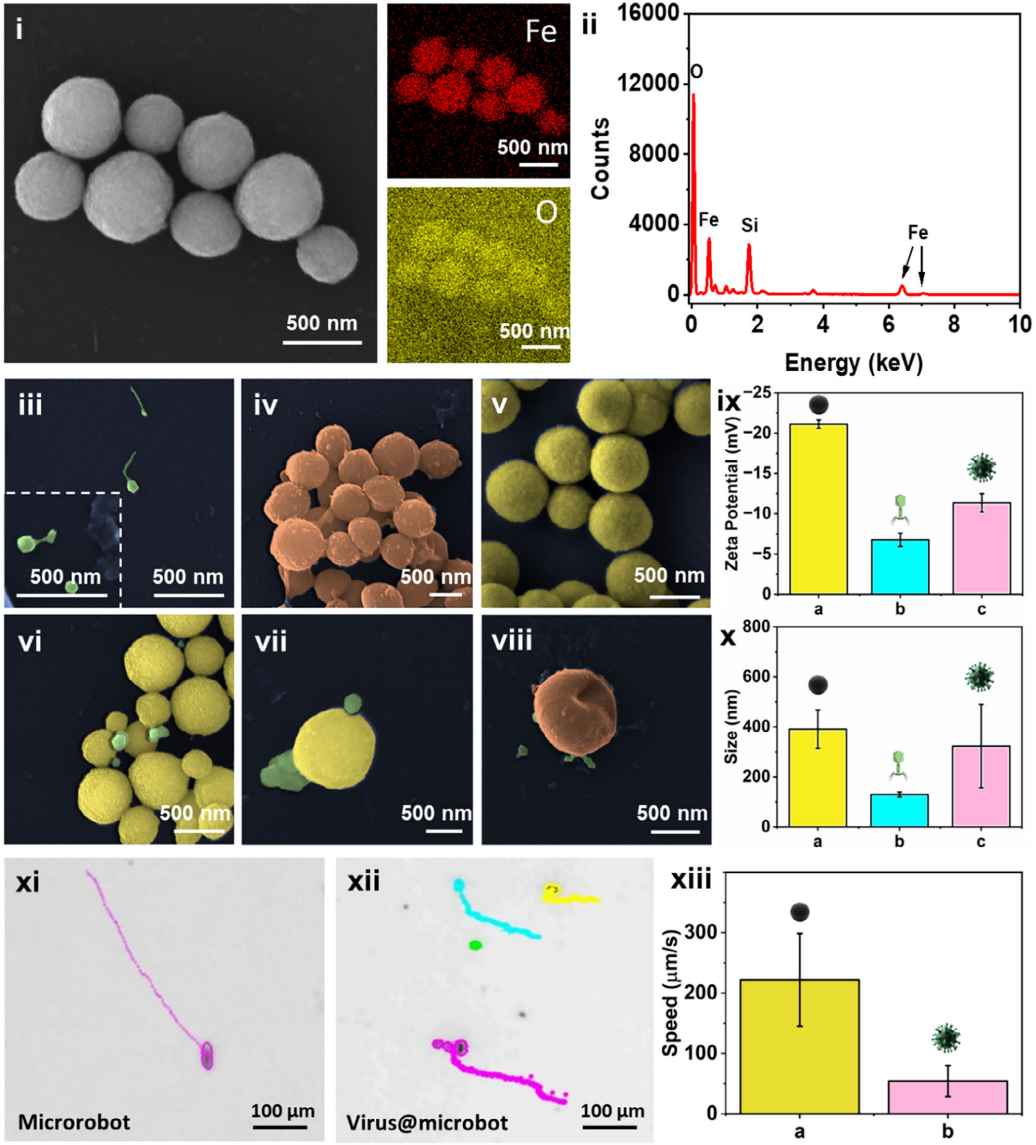
Structural, surface, and motion analysis of Fe_3_O_4_ microrobots. i) Scanning electron microscopy (SEM) image showing the spherical morphology of Fe_3_O_4_ microrobots. Elemental mapping of Fe_3_O_4_ microrobots from SEM‐EDX (Energy‐dispersive X‐ray spectroscopy) analysis, confirming the uniform distribution of Fe (red) and O (yellow). Scale bar, 500 nm. ii) EDX spectrum indicating the presence of Fe and O as primary elements in the microrobots. iii–viii) SEM images showcasing various experimental conditions: iii) isolated viruses, scale bar, 500 nm (inset: showing a magnified view of viruses, scale bar 500 nm); iv) bacteria alone, scale bar 500 nm; v) bare microrobots; vi‐vii) virus@microbots, demonstrating successful surface functionalization, scale bar 500 nm; viii) ruptured bacterial cells post‐treatment with virus@microbots. ix) Zeta potential measurements for a) microrobots, b) viruses, and c) virus@microbots, showing surface charge differences due to conjugation. x) Dynamic light scattering (DLS) measurements indicating the size distribution of a) microrobots, b) viruses, and c) virus@microbots. Figure xi‐xiii) showing the motion behaviour and magnetic control of microrobots xi), bare microrobots, showing consistent linear motion guided by the magnetic field xii) virus@microbots in the biofilm matrix. Scale bar, 100 µm. xiii) Bar chart comparing the average speeds of a) bare microrobots and b) virus@microbots under an applied magnetic field, highlighting a reduction in virus@microbots speed due to phage conjugation for the biofilm eradication. Note: SEM images are pseudo‐coloured for visual clarity.

Scanning Electron Microscopy (SEM) and Energy‐Dispersive X‐ray (EDX) analyses provided a thorough structural and elemental characterization of the Fe_3_O_4_ magnetic microrobots. These techniques provided insights into the microrobots’ morphology, chemical composition, and uniformity. SEM imaging (**Figure** **1**(i)) revealed the expected spherical shape of the Fe_3_O_4_ microrobots, a feature that supports their functionalization with viruses and facilitates magnetically guided rotatory motion and movement for biofilm elimination. To confirm their elemental composition, elemental mapping reinforced these findings by demonstrating a uniform distribution of Fe (red) and O (yellow) across the microrobots. Additionally, the EDX spectrum (Figure 1(ii)) confirms iron (Fe) and oxygen (O) as the dominant elements, consistent with the magnetite (Fe_3_O_4_) structure. The lack of impurity peaks in the spectrum highlights the high purity of the synthesized particles. This consistent spherical morphology and even elemental dispersion emphasize the reliability of Fe_3_O_4_ microrobots as a solid platform for subsequent functionalization efforts.

Additionally, supplementary structural and chemical evaluations were performed to further confirm the characteristics of the Fe_3_O_4_ microrobots. X‐ray Diffraction (XRD) analysis (Figure S1a, Supporting Information) disclosed a crystalline cubic spinel structure typical of magnetite, with diffraction peaks aligning closely with the JCPDS (Joint Committee on Powder Diffraction Standards) Card 75‐0033.^[^ ^51^, ^52^, ^53^, ^54^, ^55^ ^]^ The high crystallinity and phase purity are critical for ensuring the magnetic properties essential for microrobot functionality. Fourier Transform Infrared Spectroscopy (FTIR) analysis (Figure S1b, Supporting Information) revealed a characteristic Fe‐O stretching vibration (561 cm^−1^) and the presence of surface hydroxyl (‐OH) groups, which are essential for facilitating bacteriophage conjugation.^[^ ^54^, ^55^, ^56^, ^57^, ^58^, ^59^ ^]^ The XPS further validated the chemical composition, with wide‐scan spectra (Figure S1c, Supporting Information) confirming Fe and O as the primary components, alongside minor amounts of C. The deconvoluted XPS spectrum of Fe 2p provides detailed insights into the oxidation states of the Fe_3_O_4_ microrobots (Figure S1d, Supporting Information). The Fe 2p spectrum reveals two primary peaks at 710.8 and 724.5 eV, corresponding to Fe 2p_3/2_ and Fe 2p_1/2_, respectively, which are characteristic of magnetite (Fe_3_O_4_). The broad nature of these peaks arises from the slight chemical shift between Fe^2^⁺ and Fe^3^⁺ states present in the mixed‐valence structure of Fe_3_O_4_. Notably, the absence of the shakeup satellite peak near ≈719 eV, a distinctive feature of Fe_2_O_3_, confirms the presence of Fe_3_O_4_ rather than Fe_2_O_3_. The O1s spectrum deconvolution (Figure S1e, Supporting Information) highlights the contributions from oxide and hydroxide species on the surface. It includes metal‐oxide bonds, confirming the integrity of the Fe_3_O_4_ structure, and surface hydroxyl groups, which play a vital role in facilitating further functionalization processes. The high purity and structural integrity of the synthesized Fe_3_O_4_ microrobots.^[^ ^60^, ^61^, ^62^ ^]^ These thorough investigations show that the Fe_3_O_4_ microrobots have a strong crystalline structure, excellent chemical purity, and functional surface groups. These findings established the groundwork for the subsequent investigation.

### 2.1 Virus Conjugation with Magnetic Microrobots

After verifying the successful synthesis and structural properties of Fe_3_O_4_ microrobots, viruses were attached to their surfaces to form virus@microbots. This conjugation process aimed to merge the biological targeting precision of viruses with the mechanical adaptability of microrobots, creating a unified and effective platform for biofilm elimination. Viruses were conjugated onto the surface of Fe_3_O_4_ microrobots via noncovalent interactions, primarily driven by electrostatic attraction between the negatively charged viral capsid proteins and the hydroxyl‐rich surface of the microrobots. The conjugation was carried out by incubating the microrobots (0.1 mg mL^−1^) with viruses (10⁸ pfu mL^−1^) for 1 h at room temperature. Postconjugation, 0.05% BSA was added for 10 min to block nonspecific binding. The success of surface functionalization was confirmed through SEM images, zeta potential analysis, DLS measurements, and FTIR spectroscopy.

To examine the interactions and functionalization with biological components forming virus@microbots, SEM images were captured at different stages. Further, zeta potential analysis and DLS were also performed as shown in Figure 1. These analyses provided evidence of virus attachment and highlighted the functional advantages of immobilized viruses over free virus treatments. Figure 1(iii) shows the SEM image of isolated viruses, with the inset highlighting the capsid structure and tail fibres, confirming their structural integrity and functionality prior to microrobot attachment. Figure 1(iv) depicts the SEM of the untreated bacterial cells, showing intact morphology and the characteristic surface features of biofilm‐forming bacteria. It serves as a control to compare the after‐effects of treatment with virus@microbots. Figure 1(v) The bare Fe_3_O_4_ microrobots exhibit a uniform spherical morphology with smooth surfaces. This structural consistency facilitates effective conjugation with viruses and supports their dynamic motion under magnetic fields. Figure 1(vi–vii) SEM images of virus@microbots demonstrate successful surface functionalization, with viruses visibly attached to the microrobot surfaces. Figure 1(viii) The SEM image shows bacterial cells post‐treatment with virus@microbots, revealing disrupted cell walls and ruptured morphology. It confirms the synergistic action of virus lysis and microrobot‐induced mechanical disruption in biofilm eradication. The SEM analysis provides direct visual evidence of each stage in the preparation and application of virus@microbots, from isolated components to biofilm disruption. Further, the zeta potential measurements were performed to monitor the changes in the surface charge at each stage and confirm the virus attachments, as shown in Figure 1(ix).

Figure 1ix(a) showcases the zeta potential of bare Fe_3_O_4_ microrobots, exhibiting a negative charge (−21.11 ± 0.52 mV) reflecting the presence of surface hydroxyl (–OH) groups, which deprotonate under neutral pH to generate negatively charged ion groups.^[^ ^63^ ^]^ In contrast, isolated viruses exhibited the negative zeta potential of −6.77 ± 0.81 mV, which arises from the negatively charged capsid proteins and nucleic acids present on their surface. However, viruses also possess positively charged tail fibers, which partially offset the overall negative surface charge, resulting in a less negative potential than Fe_3_O_4_, Figure 1ix(b). After conjugation, the virus@microbots showed an intermediate zeta potential of −11.35 ± 1.13 mV, Figure 1ix(c). This change in surface charge indicates that the viruses successfully attached to the microrobot surface. The interaction is driven by noncovalent forces; mainly electrostatic attraction, hydrogen bonding, and van der Waals interactions, between the viral capsid proteins and the hydroxyl groups present on the Fe_3_O_4_ surface. As a result, the resulting composite surface exhibits a mixed population of functional groups from both the microrobot and the virus. The intermediate zeta potential observed for virus@microbots confirms that viral components have successfully interacted with and modified the microrobot surface. This shift in surface charge suggests a stable, non‐covalent association between the two components, Figure S2(i) (Supporting Information). The result aligns well with our DLS (Figure 1x; Figure S2(ii)) (Supporting Information) and FTIR analyses (Figure S3, Supporting Information), further validating that virus attachment to the microrobots was achieved effectively.^[^ ^42^, ^64^, ^65^, ^66^ ^]^ Furthermore, Dynamic light scattering (DLS) measurements (Figure 1(x)) provided additional evidence of successful virus conjugation by showing a clear increase in hydrodynamic size after surface functionalization. Bare Fe_3_O_4_ microrobots exhibited a relatively smaller hydrodynamic diameter due to their compact, unmodified structure. In contrast, virus@microbots displayed a larger average size, consistent with the attachment of bacteriophages onto the microrobot surface. The consistent size distribution indicates that virus conjugation did not cause particle aggregation, suggesting good colloidal stability. To further confirm this trend, Figure S2(ii) (Supporting Information) presents the hydrodynamic size distribution for microrobots, viruses, and virus@microbots across varying virus concentrations, while maintaining the microrobot concentration constant at 0.1 mg mL^−1^. As expected, the hydrodynamic diameter of virus@microbots increased proportionally with viral loading, supporting the concentration‐dependent surface binding and supporting the conjugation efficiency observed in zeta potential.

Further confirmation of virus conjugation was obtained through FTIR analysis, as shown in Figure S3 (Supporting Information). Spectrum (a) corresponds to the bare Fe_3_O_4_ microrobots, showing characteristic Fe–O stretching vibrations near ≈580 cm^−1^ and a broad peak ≈3000 cm^−1^ attributed to surface hydroxyl (—OH) groups. After conjugation with viruses, spectrum (b) displays additional peaks in the ≈3000–3200 cm^−1^ range (N─H stretching) and ≈1500–1700 cm^−1^ range (C═O stretching), which are typical of amide bond formation. These new signals indicate the presence of proteinaceous viral components on the microrobot surface, confirming successful virus immobilization. The retention of the Fe─O peak further confirms that the Fe_3_O_4_ core remains structurally intact after functionalization.

Hence, this functionalization of viruses onto Fe_3_O_4_ microrobots provided a significant benefit over free viruses, allowing for a thorough method of biofilm removal. Attaching viruses to microrobots allows them to physically penetrate the biofilm structure more effectively, assisted by the magnetic propulsion of the Fe_3_O_4_ core. This mechano‐biological synergy enhances the ability of viruses to reach and act on deeply embedded bacteria.^[^ ^49^, ^67^ ^]^ Additionally, this functionalization guarantees localized high virus concentrations,^[^ ^30^ ^]^ which overcomes a significant drawback of free viruses, which tend to dilute in bulk media. This localized concentration increases therapeutic efficacy while also protecting the viruses from degradation by environmental enzymes like proteases. As a result, the functionalized viruses remain active longer and retain their infectivity.^[^ ^68^, ^69^ ^]^ Moreover, magnetic guidance enables precise delivery of virus@microbots within biofilm matrices, reducing off‐target effects and improving treatment accuracy.^[^ ^67^, ^70^ ^]^ Altogether, these combined features make virus@microbots a novel and versatile tool for tackling persistent biofilm‐associated infections.

### 2.2 Motion Behaviour and Magnetic Control of Microrobots

The motion dynamics and magnetic responsiveness of bare microrobots and virus@microbots were investigated to determine their effectiveness in targeted biofilm disruption. Trajectory tracking (Figure 1(xi),(xii)) further revealed that bare microrobots followed linear paths in non‐biofilm settings with little resistance, while virus@microbots showed slightly altered paths in biofilms, indicative of matrix disruption. In Figure 1(xiii), average speed analysis indicated that bare microrobots, with smoother surfaces, achieved higher velocities (221 ± 76.96 µm ^−1^s), whereas virus@microbots exhibited a slower speed of 54 ± 25.61 µm ^−1^s, nearly four times less. This reduction results from increased hydrodynamic drag caused by the attached viruses and the physical resistance of the dense biofilm environment. Importantly, this slower movement is not a limitation; instead, it reflects active mechanical engagement with the biofilm and the simultaneous biological lysis of bacteria by the surface‐bound viruses. Despite reduced speed, virus@microbots retained sufficient mobility and responsiveness to the external magnetic fields, supporting their robustness and adaptability for precise biofilm eradication through integrated magnetic and biological mechanisms.

This behavior is further demonstrated in Videos S1–S3 (Supplementary Video1 to Video3). Video S1 (Supplementary Video1) shows the smooth, linear motion of bare microrobots under magnetic actuation. Videos S2 and S3 (Supplementary Video2 and Video3) highlight virus@microbots moving with reduced velocity and altered paths, indicating interactions with bacterial cells. In particular, Video S3 (Supplementary Video3) captures virus@microbots navigating within a biofilm, where disrupted trajectories and slowed motion illustrate their active penetration, bacterial capture, and matrix engagement in real time.

### 2.3 Biofilm Eradication Efficacy

#### 2.3.1 Optimization of Virus Concentration and Incubation Time

The effectiveness of virus@microbots in biofilm eradication was evaluated through optimization studies and comparative analyses focused on virus concentration, incubation time, and treatment conditions (static vs dynamic). These investigations employed quantitative biofilm mapping and crystal violet staining to systematically assess performance under varying conditions. To begin, the virus concentration was optimized while keeping the microrobot concentration constant at 0.1 mg mL^−1^. Virus concentrations were varied from 10^4^ to 10^10^ pfu mL^−1^. As shown in Figure S4(i) (Supporting Information), increasing the virus concentration (from 10^4^, 10^6^, 10^8^, 10^9^, 10^10^ pfu mL^−1^) led to progressively improved biofilm removal, with 10^10 ^pfu mL^−1^ achieving the highest eradication efficiency and leaving minimal residual biofilm. However, 10^8^ pfumL^−1^ was selected as the optimal concentration for subsequent studies, as it demonstrated intermediate efficacy while preserving viral activity and avoiding saturation effects.

Next, the impact of incubation time was examined under optimized conditions (microrobots 0.1 mg mL^−1^, viruses 10^8^ pfu mL^−1^), as shown in Figure S4(ii) (Supporting Information). The biofilm eradication was monitored from 0 to 24 h, with the results represented as mean values from three independent measurements, accompanied by standard deviation error bars. A time‐dependent increase in biofilm removal was observed. During the initial phase (0–6 h), biofilm removal progressed rapidly, with eradication efficiency increasing to 48.21% at 1 h, 64.03% at 3 h, and peaking at 65.77% at 6 h. This fast response reflects the active role of virus@microbots in penetrating the biofilm and delivering lytic viruses to bacterial targets. Beyond 6 h, the biofilm eradication rate saturated with marginal increases; 74.37% at 9 h, 74.53% at 12 h, and stabilized at 74.67% at 24 h. The saturation indicates either the exhaustion of available bacterial targets or a reduced level of residual viral activity within the dense matrix. These findings collectively established that 10^8^ pfu mL^−1^ virus concentration and 6 h incubation are the optimal parameters for biofilm treatment using virus@microbots. Further, Figure S5 (Supporting Information) displays microplate images from crystal violet staining used to evaluate the effectiveness of virus@microbots by highlighting differences in residual biomass across virus concentrations and time points.

Similarly, Figure S6 (Supporting Information) provides biofilm mapping images that visually support the optimization trends and effectiveness of virus@microbots in biofilm disruption under the tested conditions.

#### 2.3.2 Targeted Biofilm Eradication Using Virus@microbots

**Figure 2:**
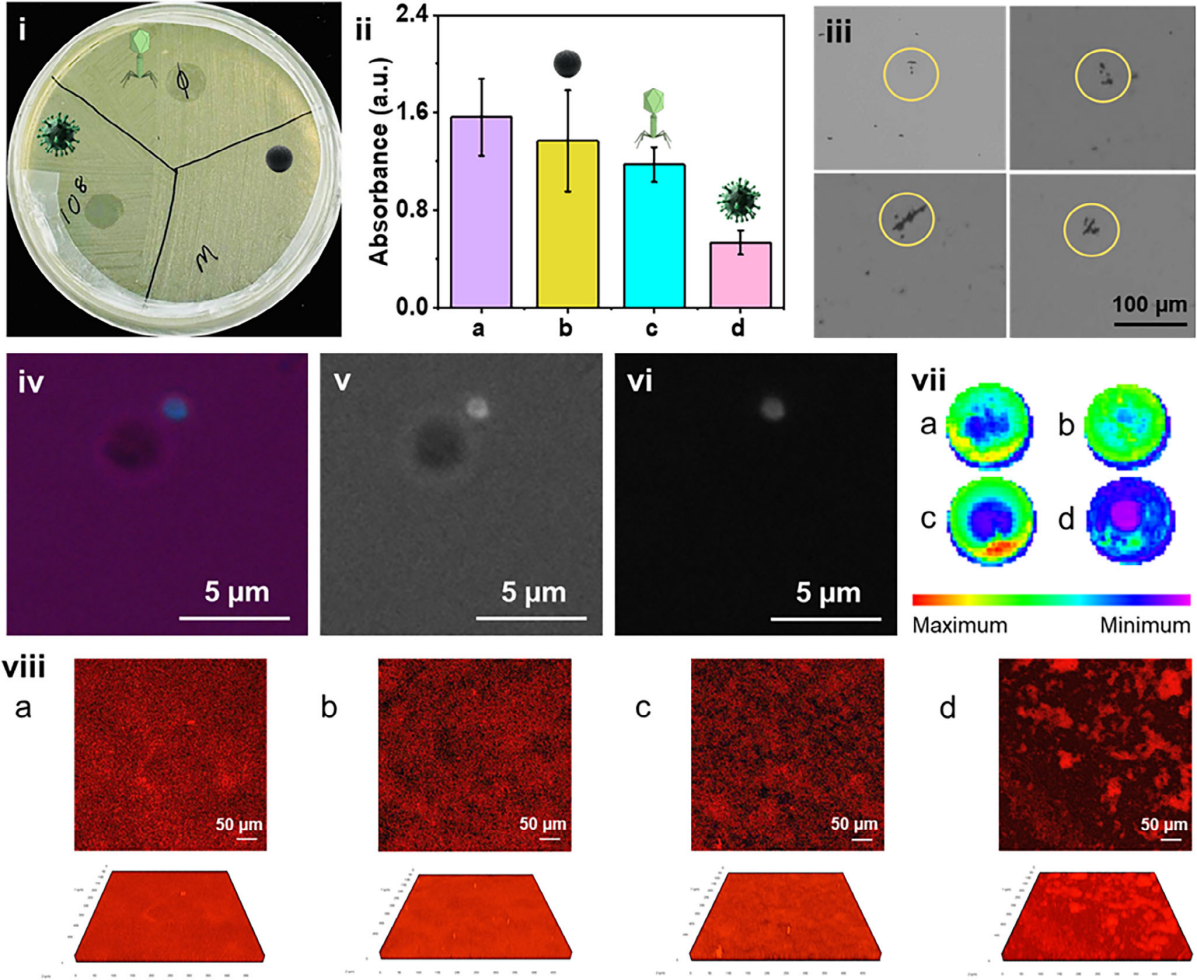
Targeted biofilm eradication using virus@microbots i) Biofilm disruption by virus@microbots under static conditions. Diameter of Petri dish 9 cm. As inset images of the virus, root, and conjugate for better visualization. ii) Tracking images of biofilm eradication by virus@microbots a) blank control b) microrobots only c) viruses only d) virus@microbots iii) Inverted microscopy images illustrating magnetic trapping of bacteria by virus@microbots under a magnetic field. iv‐vi) Fluorescence microscopy images showing the virus@microbots bacteria interaction: iv) multichannel mode, v) brightfield mode, and vi) fluorescent mode. vii) Biofilm mapping analysis where maximum to minimum on the scale indicates the relative density or amount of residual biofilm present in each treatment condition: a) blank control, b) microrobots‐only control, c) virus‐only control, and d) virus@microbots. Diameter of well: 6.2 mm. viii) showing the 2D‐images along with their 3D reconstituted images of the SYPRO RUBY stain under various conditions a) biofilm control b) virus control c)microrobot control d) virus@microbots. Scale bar used = 50 µm; Z axis thickness  =  23.76 µm. Experimental Conditions: Virus concentration: 10⁸ pfu mL^−1^; microrobot concentration: 0.1 mg mL^−1^; treatment time: 6 h; magnetic field: 5 mT.

**Figure** **2** presents a comprehensive evaluation of the biofilm removal performance of virus@microbots under optimized conditions (virus concentration 10⁸ pfu mL^−1^, microrobot concentration 0.1 mg mL^−1^, treatment duration 6 h, magnetic field strength 5 mT). This figure integrates real‐time motion tracking, quantitative absorbance measurements, fluorescence microscopy, and biofilm mapping to illustrate the targeted dismantling of biofilms by virus@microbots. Figure 2(i–ii) compares biofilm removal under static and dynamic conditions. Under *static* conditions, virus@microbots entirely depend on passive diffusion, resulting in limited biofilm removal, as shown by the residual biofilm in Figure 2(i). Conversely, under magnetic actuation (Figure 2(ii)), the active motion of virus@microbots significantly enhances penetration into the biofilm and delivers viruses effectively. Thus, improving biofilm eradication. The corresponding quantitative data in Figure 2ii(a–d) reflect the eradication efficiency of different treatments based on absorbance after crystal violet staining. Figure 2ii(a) displays the absorbance of untreated biofilms (blank control), serving as a baseline. Bare microrobots alone (Figure 2ii(b)) have reduced absorbance and achieved an eradication efficiency of 12.37%, due to modest mechanical disruption. Treatment with free viruses, shown in Figure 2ii(c), resulted in 24.77% eradication, showing moderate bacterial lysis but limited matrix penetration. In comparison, virus@microbots Figure 2ii(d) achieved 65.77% removal, highlighting their superior performance and demonstrating a synergistic improvement by combining mechanical disruption with targeted viral killing. Figure 2(iii) highlights the ability of virus@microbots to physically trap and interact with bacterial aggregates under a magnetic field, as captured by inverted microscopy. The images emphasize the importance of magnetic guidance in enabling microrobots to target and break up bacterial clusters within the biofilm. Figure 2(iv)‐(vi) presents fluorescence microscopy images that further showcase the mechanisms of biofilm disruption. In Figure 2(iv), multichannel mode overlays fluorescence and brightfield signals, revealing real‐time interactions between virus@microbots and bacterial cells within the biofilm. This image demonstrates how the microrobots navigate through the matrix and position themselves for effective viral delivery. Figure 2(v) shows the brightfield mode, which offers a clear view of the spatial distribution of virus@microbots relative to the biofilm structure, confirming their proximity and targeted motion under magnetic guidance. In Figure 2(vi), the fluorescence mode shows the distribution and interaction of virus@microbots with the biofilm matrix. Increased fluorescence signal around bacterial clusters indicates active engagement by virus@microbots, helping to visualize their localization and activity within the biofilm structure. Additional confirmation of bacterial targeting is shown in Figure S6 (Supporting Information), where fluorescence microscopy reveals the virus@microbots’ ability to trap bacteria. Images in brightfield, fluorescent, and multichannel modes (Figure S6(i–ii) (Supporting Information) demonstrate their active engagement with bacterial cells under optimized conditions. These findings align with earlier results (Figure 2(iv‐vi)), reinforcing that virus@microbots disrupt biofilms through a combination of physical motion and biologically driven lysis.

#### 2.3.3 Biofilm Mapping for Optimization of Virus Concentration and Incubation Time

To strengthen our findings, we also performed comparative biofilm mapping under various treatments. This approach provided a clear visual representation of biofilm density, where signal intensity indicated biofilm removal. Figure 2vii(a‐d) displays comparative biofilm mapping data for all treatment conditions. Figure 2vii(a) represents a blank control, i.e., untreated biofilm showing high biofilm density. The microrobot control only (Figure 2vii(b)) shows minimal reduction in biofilm density, highlighting that mechanical action alone is insufficient. Figure 2vii(c) virus control only shows partial removal, reflecting limited penetration by free viruses. It highlights the importance of an active delivery system to improve virus penetration. In contrast, Figure 2vii(d) displays the biofilm mapping data for virus@microbots under dynamic conditions, exhibiting the most significant matrix clearance with minimal residual biofilm, validating the synergistic efficiency of this *dual‐action* system. Magnetic actuation ensures targeted movement, while virus activity provides localized bacterial eradication. Further, the optimization parameters were validated by using the biofilm mapping, as shown in Figure S7 (Supporting Information). In Figure S7(i) (Supporting Information), varying the virus concentration while keeping microrobot levels constant demonstrated a clear concentration‐dependent reduction in biofilm signal. Similarly, FigureS7(ii) (Supporting Information) shows time‐dependent eradication efficiency, with maximum effect observed after 6 h of incubation. These visual trends closely align with the quantitative results presented in Figures S4 and S5 (Supporting Information), further supporting the chosen conditions for effective biofilm disruption.

Next, to assess the structural integrity of the biofilm matrix, we performed SYPRO Ruby staining, which specifically binds to the proteinaceous components of the extracellular polymeric substances (EPS). This allowed us to visually compare the extent of matrix disruption across different experimental groups. As shown in Figure 2(viii), the untreated biofilm control Figure 2viii(a) exhibited strong SYPRO Ruby fluorescence, indicating a well‐developed, protein‐rich matrix. Similarly, both the virus‐only Figure 2viii(b) and microrobot‐only Figure 2viii(c) treated groups retained high signal intensity, suggesting minimal disruption of the EPS structure when applied independently. In contrast, the virus@microbots group Figure 2viii(d) showed a marked decrease in fluorescence intensity, indicating substantial degradation of the protein matrix. This reduced signal reflects the effective action of virus@microbots in breaking down biofilm architecture through a combined mechanical (microrobot‐driven disruption) and biological (phage‐mediated lysis) approach. These results confirm that virus@microbots are capable not only of targeting bacterial cells but also of dismantling the protective protein scaffolding of biofilms, offering a robust and multifaceted strategy for biofilm eradication.

### 2.4 Live/Dead Fluorescence Assay of Biofilm Viability

**Figure 3:**
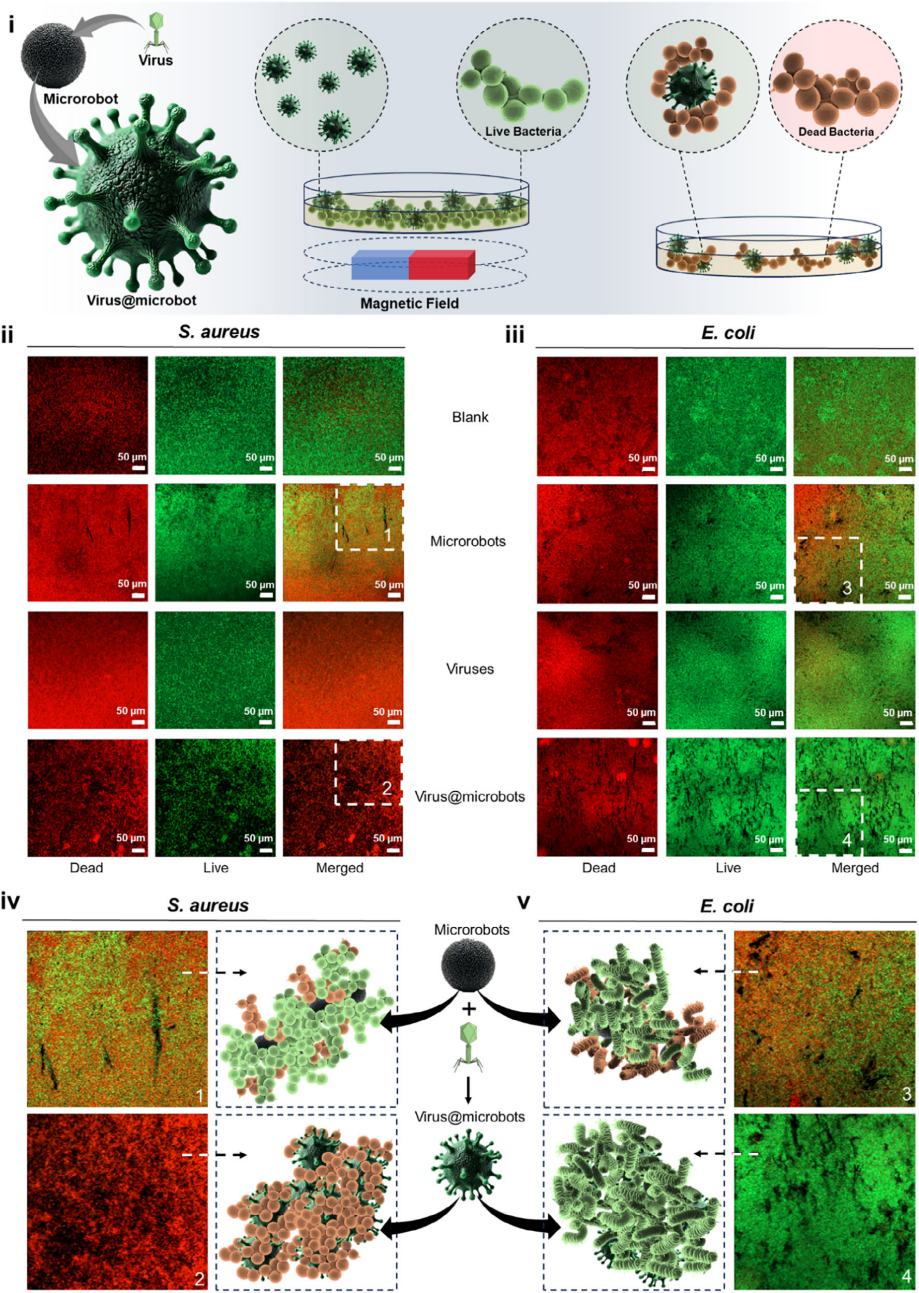
Confocal microscopy images of the Live/Dead assay. i) Schematic representation of virus@microbots showing the magnetic guidance, targeted interaction with bacterial biofilms, and their *dual‐action* mechanism; virus‐mediated lysis and microrobot‐induced disruption. ii) & iii) Confocal Live/Dead microscopy images showing bacterial viability across treatment groups (blank control, microrobots only, viruses only, and virus@microbots). ii) Treatment against *S. aureus* biofilms demonstrates strong red fluorescence in the virus@microbot group, indicating extensive bacterial killing due to virus selectivity and microrobot penetration. iii) In contrast, *E. coli* biofilms show minimal red signal in the virus@microbot group, validating the strain specificity of the viruses used, which target *S. aureus* but not *E. coli*. The observed biofilm disruption in *E. coli* is primarily due to the mechanical motion of microrobots. Scale bars of 50 µm for both *ii* and *iii*. iv) & v) Zoomed‐in confocal images and schematic representations of microrobot interaction in (see inset 1,3) control and (see inset 2,4) virus@microbot‐treated groups for *S. aureus* and *E. coli*, respectively, highlighting bacterial capture and structural disruption. Green  =  live cells (SYTO9); red  =  dead cells (PI). Experimental Conditions: Virus concentration  =  10⁸ pfu mL^−1^; microrobot concentration  =  0.1 mg mL^−1^; treatment time = 6 h, scale bar in (ii & iii) = 50 µm, size of insets (1–4  =  275 × 275 µm).

**Figure** **3**(i) presents a schematic overview of the virus@microrobot system and its mechanism for biofilm eradication. It illustrates how viruses are conjugated onto the surface of Fe_3_O_4_ microrobots and magnetically guided toward bacterial biofilms. Once at the target site, the microrobots actively penetrate the biofilm matrix, where their combined mechanical motion and virus‐induced lysis facilitate localized bacterial killing and biofilm disruption. To visually confirm the bactericidal effect, biofilm disruption, and bacterial selectivity of the virus@microrobots, we performed a Live/Dead staining assay using SYTO9 (green, viable cells) and propidium iodide (PI; red, membrane‐compromised dead cells), followed by confocal laser scanning microscopy. This enabled visualization of bacterial viability within the biofilm of *S. aureus* post‐treatment. As shown in Figure 3(ii), the untreated *S. aureus* biofilms (blank control) predominantly exhibited green fluorescence, indicating a healthy and intact population. Samples treated with microrobots alone or free viruses showed only a slight increase in red fluorescence, reflecting a limited bactericidal effect. In contrast, virus@microrobot‐treated biofilms displayed a dominant red fluorescence and a marked reduction in green fluorescence; clear evidence of effective bactericidal potential driven by both virus‐mediated lysis and microrobot‐assisted biofilm disruption.

To evaluate specificity, the same assay was applied to Escherichia coli (*E. coli*) biofilms (Figure 3(iii)). Since the viruses used in this study are selective for Gram‐positive *S. aureus*, the virus@microrobots showed no significant effect on *E. coli*. The biofilms retained strong green fluorescence with minimal red signal, similar to the untreated control. This outcome confirms the selective nature of the virus@microrobots and highlights their potential for targeted bacterial therapy with minimal off‐target impact. High‐magnification images in Figure 3(iv),(v) further validate this result. In *S. aureus*, virus@microrobots clearly showed bactericidal effect and disrupted the biofilm structure, whereas E. coli biofilms remained largely unaffected. The red fluorescence in only microrobots‐treated *S. aureus* and *E. coli* is indicative of the reactive oxygen species‐mediated bactericidal effect.^[^ ^71^ ^]^ These findings demonstrate the pathogen‐specific precision of the virus@microrobots and underscore their promise for targeted antimicrobial applications.

### 2.5 Validation of Virus@Microrobot Antibiofilm *Efficacy* on Real Samples

**Figure 4:**
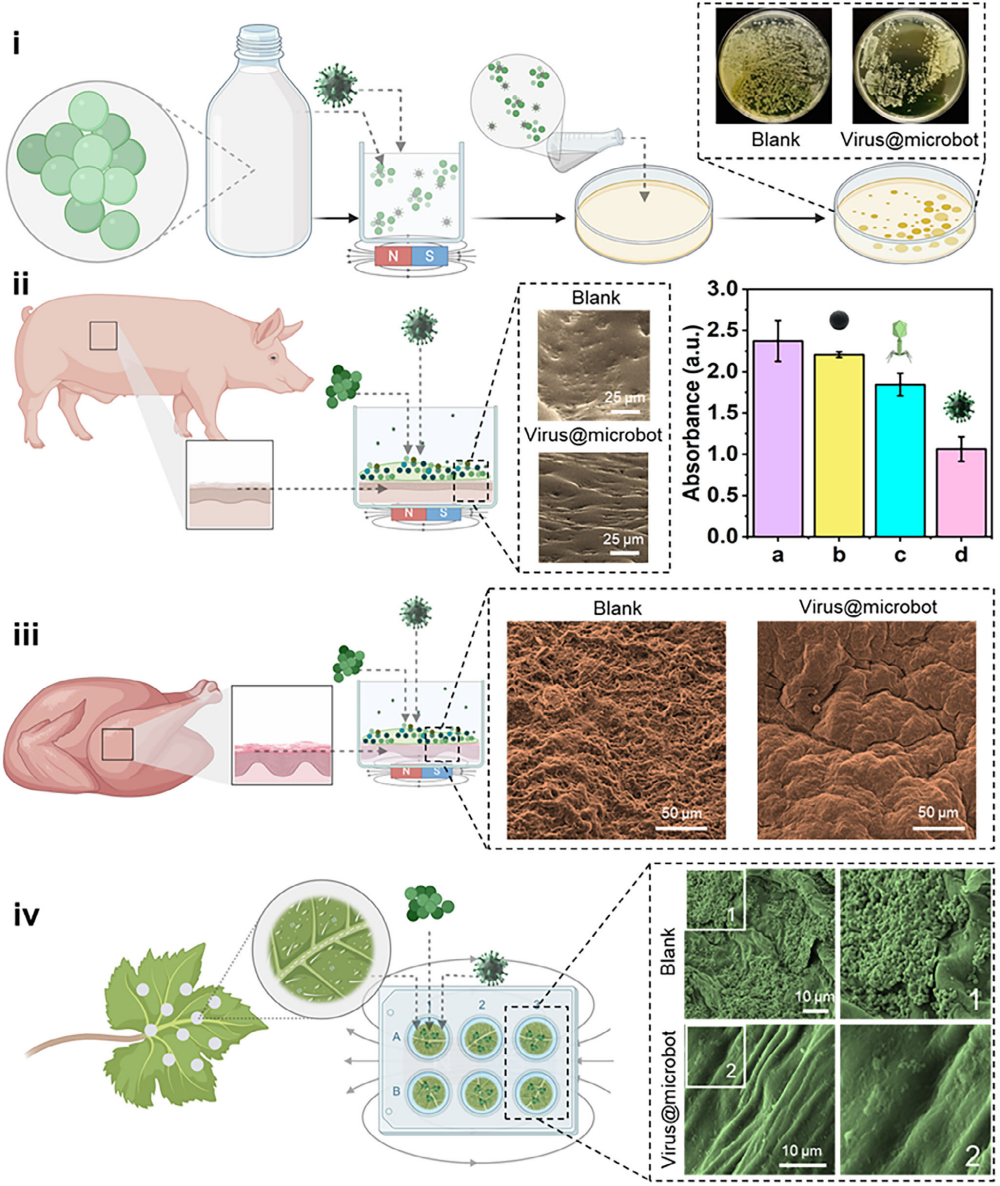
Validation of virus@microbots for bacteria/biofilm eradication in biofluid/on diverse biological surfaces. To demonstrate the practical applicability of virus@microbots, biofilms were cultured for 24 h on various representative surfaces, including milk, mammalian skin, avian skin, and plant leaves, and subsequently rinsed with 1× PBS to remove non‐adherent biomass. i) Agar plating results from milk samples inoculated with *S. aureus* biofilms, comparing untreated (blank control) and virus@microbots‐treated groups. Clear reduction in bacterial colonies confirms effective biofilm disruption in complex food matrices under static conditions. Diameter of Petri dish 9 cm. ii) SEM images of porcine skin show dense biofilm in the untreated control and significant surface cleaning after virus@microbot treatment. The crystal violet assay supports these findings, with absorbance data highlighting stepwise biofilm reduction across treatments: a) blank control, b) microrobots only, c) viruses only, and d) virus@microbots. iii) SEM images of chicken skin before and after virus@microbot application demonstrate effective biofilm clearance, indicating suitability for poultry‐related surfaces. iv) SEM analysis of maple leaf surfaces before (image 1) and after (image 2) treatment. In the control, biofilms are visibly dispersed over the surface, while the treated sample shows notable disruption and removal of biofilm structures, confirming applicability in plant‐based systems. Experimental Conditions: Virus concentration  =  10⁸ pfu mL^−1^; microrobot concentration  =  0.1 mg mL^−1^; treatment time  =  6 h.

To demonstrate the broader practical relevance of virus@microbots, we evaluated their antibiofilm activity on a variety of “biological substrates, ranging from biofluid, plant leaf, and animal skin”. These models represent diverse environments frequently impacted by bacteria and biofilms (**Figure** **4**).

First, commercial milk was chosen as a representative nutrient‐rich, proteinaceous biofluid prone to microbial contamination. *S. aureus* was incubated in milk for over 4 h. The samples were then incubated with virus@microbots under a magnetic field for 2 h, and then the milk was spread onto the agar plates. As shown in Figure 4(i), a clear reduction in colony formation was observed in treated samples compared to the untreated control, confirming bacterial inhibition even in an opaque, complex fluid like milk. To further validate the interaction of virus@microbots within this medium, FTIR spectroscopy was performed and presented in Figure S8 (Supporting Information): Spectrum (a) shows milk alone, displaying a broad band at 3300–3400 cm^−1^ corresponding to hydrogen‐bonded —OH and —NH groups, characteristic of proteins and water content in milk. Spectrum (b), representing virus@microbots in milk, shows more defined peaks at ≈1650 and ≈1550 cm^−1^, corresponding to Amide I and Amide II vibrations, confirming the presence of protein structures on the microrobots. Additional peaks near 1050 cm^−1^ suggest C─O or P═O stretching, indicative of successful surface bioconjugation. Spectrum (c), for virus@microbots in water, exhibits lower absorbance and fewer defined bands, reflecting the absence of the complex milk matrix; though weak signals from Fe–O or residual protein components may still be detected. These FTIR results further confirm successful virus conjugation and demonstrate that virus@microbots retain their structural fingerprint in both biological and aqueous environments.

To evaluate virus@microbot performance on tissue surfaces, ex vivo porcine skin, a physiologically relevant mammalian model, was selected. After biofilm formation and treatment, the samples were fixed and dehydrated for SEM imaging. The SEM revealed a cleaner skin surface post virus@microbot treatment compared to the visibly spiked and disturbed surface in the untreated control (Figure 4(ii)). In parallel, a crystal violet assay quantified residual biofilm levels on the porcine skin surface post treatment. A significant reduction in absorbance was observed in the virus@microbots(d), compared to the blank (a), microrobots‐only (b), and virus‐only (c) treatments. These results underscore the enhanced efficacy of virus@microbots. We further explored chicken skin as an avian model, relevant in food processing and hygiene contexts. SEM analysis (Figure 4(iii)) showed substantial biofilm clearance in the virus@microbots treated samples, suggesting that they could be explored for biofilm control in poultry‐related antimicrobial research.

At last, the maple leaf surface was also examined for virus@microbot performance, as leaves are critical yet often overlooked post‐harvest surfaces prone to bacterial growth. After 24 h of biofilm growth and subsequent treatment, SEM images revealed dense microbial coverage in the untreated control *(image 1*), while the treated surface *(image 2)* showed pronounced biofilm removal (Figure 4(iv)). Across all these models, experimental conditions were kept consistent; virus concentration of 10⁸ pfu mL^−1^, microrobot concentration of 0.1 mg mL^−1^, and treatment duration of 6 h. Together, these findings highlight the versatility and practical relevance of virus@microbots in tackling biofilms across diverse biological surfaces, demonstrating their promise for gradual translation from lab‐scale systems to real‐world applications in healthcare, food, and agriculture.

## 3 Conclusion

This study demonstrates the development and application of virus@microbots as an innovative and effective strategy for biofilm eradication. By harnessing the biological specificity of viruses and the mechanical precision of Fe_3_O_4_ microrobots, virus@microbots offer a *dual‐action* approach; mechanical disruption of biofilm matrices and localized bacterial lysis. These combined functionalities render virus@microbots highly versatile for a wide range of applications. This synergy enables targeted, efficient biofilm clearance, offering broad applicability across biomedical and industrial domains. In clinical settings, virus@microbots could be deployed to combat persistent infections on catheters, implants, and other medical devices. In industrial environments, they hold potential for reducing biofilm‐associated fouling in pipelines, food processing surfaces, and water treatment systems.

In the future, the focus should be on scaling up the production of virus@microbots for real‐world applications and testing their performance against a broader spectrum of biofilm‐forming pathogens. Moreover, investigations into the long‐term stability, biocompatibility, and environmental safety of virus@microbots are also warranted. Additionally, integrating intelligent sensing or responsive functionalities into the microrobots could enhance their efficiency and versatility. Virus@microbots represent a promising, scalable solution to address biofilm‐associated challenges across diverse fields, bridging the gap between advanced antimicrobial strategies and practical implementation.

## 4 Experimental Section

### Synthesis of Magnetite (Fe_3_O_4_) Particles

Magnetite magnetic microparticles were synthesized by using a one‐step hydrothermal method. In detail, 2.5 mmol of FeCl_3_·6H_2_O is mixed in 20 mL of ethylene glycol, after by the addition of 1.8 g of sodium acetate and 0.5 g of polyethylene glycol (PEG) as a surfactant. The mixture was left for continuous stirring for 30 min to 1 h until a transparent solution was achieved. The resulting solution was later transferred into a 50 mL Teflon‐lined stainless‐steel autoclave and heated at 200 °C for 12 h. After heating, the autoclave was left at room temperature for natural cooling. Later, the product was thoroughly washed several times with deionized (DI) water, ethanol, and collected by centrifugation. Finally, the purified microparticles were dried in a vacuum oven at 60 °C for 6 h.

### Microrobots Morphological, Chemical, & Structural Characterization

The characterization of magnetite microparticles was performed using a range of advanced techniques. Scanning Electron Microscopy (SEM) was used to take images by using a Tescan MIRA 3 XMU instrument, which provides detailed insights about the morphology of the particles. Energy‐dispersive X‐ray Spectroscopy (EDX) mapping was performed with an Oxford EDX detector attached to the SEM, enabling elemental distribution analysis. Further, X‐ray Diffraction (XRD) patterns were studied by using a Rigaku SmartLab 3 kW diffractometer equipped with a fine‐focus Cu‐sealed tube, operating at 40 kV and 30 mA, to examine the crystalline structure of the microparticles. To know the atomic composition of the samples, X‐ray Photoelectron Spectroscopy (XPS) using an AXIS Supra instrument (Kratos Analytical, Japan) with a monochromatic Al Kα (1486.7 eV) excitation source was used. Casa XPS software is used to fit spectral fitting and analysis. Zeta potential measurements were conducted using a Malvern Zetasizer to evaluate the charge distribution and dynamic light scattering (DLS) properties of the microrobots. To further analyze the chemical bonding within the samples, Fourier Transform Infrared Spectroscopy (FTIR) measurements were recorded using a Vacuum FTIR Vertex 70v (Bruker, USA) over a spectral range of 4000–400 cm^−1^. These comprehensive analyses provided detailed structural, chemical, and physical insights into the magnetite microrobots.

### Motion Studies

To record the microrobot's motion, a Nikon ECLIPSE TS2R inverted microscope coupled with a Basler digital camera acA1920‐155uc was used. In detail, the microrobots suspended in water were dropped on a glass slide. Videos were recorded at 20 fps and analysed using NIS Elements Advanced Research software to track microrobot movements and calculate their speed.

### Bacterial Strains, Viruses, and Cultural Conditions

*S. aureus* was purchased commercially and propagated on a strain grown in meat peptone broth (MPB) medium at 37 °C. Viruses were also obtained commercially (ATCC, *USA*) and stored at 4 °C in buffer, comprising 50 mM Tris‐HCl (pH 7.5), 0.1 mM NaCl, 8 mM MgSO_4_, and 0.01% gelatin to maintain stability and activity.^[^ ^72^ ^]^ The virus titer was determined using a double‐layer agar method and expressed as plaque‐forming units (pfu) per millilitre. All culturing and handling procedures were conducted in aseptic conditions to prevent contamination and ensure reproducibility.

### Viruses Conjugation with Magnetic Microrobots

To conjugate the viruses on the surface of the Fe_3_O_4_ microrobots, the Fe_3_O_4_ microparticles, synthesized via the hydrothermal method and thoroughly washed, were directly dispersed in phosphate‐buffered saline (PBS) to form a stable suspension. Purified viruses, suspended in buffer, were gently mixed with the activated Fe_3_O_4_ microrobots suspension in a controlled ratio to ensure optimal binding. The reaction mixture was incubated at room temperature with gentle rotation for 2 h, allowing the amino and carboxyl groups on the virus capsid proteins to interact with the Fe_3_O_4_ surface naturally. The electrostatic, hydrophobic interactions, and Van der Waals forces govern the binding of viruses to Fe_3_O_4_ microparticles.^[^ ^42^, ^67^, ^73^ ^]^ These interactions facilitated stable attachment without requiring additional surface activation steps. The virus‐bound microparticles were treated with 0.05% bovine serum albumin (BSA) in PBS for 10 min to minimize nonspecific interactions and stabilize the conjugates.^[^ ^30^ ^]^

Excess viruses and unbound proteins were removed by repeated washing with PBS, ensuring the purity of the virus‐conjugated Fe_3_O_4_ particles. The successful conjugation of viruses onto the Fe_3_O_4_ microrobots was confirmed using Zeta Potential Measurements, Fourier Transform Infrared Spectroscopy (FTIR), SEM & Dynamic Light Scattering (DLS).

### Experimental Setup for Biofilm Cultivation and Eradication

Biofilms were established by inoculating bacterial strains into a nutrient‐enriched medium optimized for biofilm formation. Specifically, a suspension of *S. aureus* was adjusted to an optical density of 0.5 at 600 nm (OD_600_) to standardize bacterial concentration. This suspension was then aliquoted into sterile 96‐well plates, with each well receiving a uniform volume (e.g., 200 µL) to ensure consistent bacterial distribution, and incubated at 37 °C for 24 h to facilitate biofilm maturation. Postincubation, the biofilms were gently washed with phosphate‐buffered saline (PBS) to remove non‐adherent cells, preserving the structurally intact biofilm matrix for subsequent eradication experiments. The uniformity and robustness of the biofilms were confirmed and quantified through crystal violet staining, validating their suitability and reproducibility for downstream eradication studies.

### Live/Dead Assay Experiments

To evaluate bacterial viability and validate biofilm disruption post‐treatment, a Live/Dead staining assay was performed using the FilmTracer LIVE/DEAD Biofilm Viability Kit (Invitrogen, Thermo Fisher Scientific; Catalog No. L10316). This two‐color fluorescence assay differentiates live and dead bacteria based on membrane integrity. The kit contains SYTO9 green fluorescent nucleic acid stain, which penetrates intact bacterial membranes, and propidium iodide (PI), a red‐fluorescent stain that enters only bacteria with compromised membranes.

Biofilms of *S. aureus* and *E. coli* were cultivated in 96‐well plates for 24 h. After treatment with respective agents (bare microrobots, free viruses, or virus@microrobots), wells were gently rinsed with sterile phosphate‐buffered saline (PBS) to remove non‐adherent cells. The wells were then stained with SYTO9 and PI according to the manufacturer's protocol and incubated in the dark at room temperature (≈25 °C) for 15 min. After washing with PBS, the stained samples were immediately imaged. *Note*: The fluorescent staining was performed according to the manufacturer's protocol.

Confocal laser scanning microscopy was performed using a Zeiss LSM900 microscope equipped with an Airyscan2 detector (ZEISS, Oberkochen, Germany). SYTO9 and PI fluorescence were recorded using standard laser settings with excitation/emission wavelengths of for SYTO9 and for PI. Imaging parameters remained constant across all samples. Image analysis was conducted using Zeiss software (ZEN Blue), and experiments were performed in triplicate to ensure reproducibility.

### SYPRO Ruby Biofilm Matrix Staining

To visualize and compare the structural integrity of the extracellular polymeric matrix (EPM) in bacterial biofilms after treatment, we employed SYPRO Ruby Protein Gel Stain (Supelco, Merck; Catalog No. S4942‐200ML). This ultrasensitive, ready‐to‐use luminescent stain selectively binds to a wide range of biofilm‐associated proteins, allowing quantification and imaging of biofilm protein content.

Biofilms of *S. aureus* were cultured in 96‐well plates for 24 h and then treated with either microrobots, free viruses, or virus@microrobots. Following treatment, wells were gently washed with sterile PBS to remove loosely attached biomass and stained with SYPRO Ruby for 30 min in the dark at room temperature (≈25 °C). Samples were rinsed again with PBS to remove unbound stain.

Fluorescence imaging was performed using a Zeiss LSM900 confocal microscope equipped with Airyscan2 (ZEISS, Oberkochen, Germany), using an excitation wavelength of 450 nm and an emission wavelength of 610 nm. Consistent acquisition settings were used for all experimental groups. Image analysis and quantification were carried out using Zeiss ZEN Blue software. All experiments were conducted in biological triplicate to ensure statistical reliability.

### Preparation of Real Samples for Biofilm Assays and SEM Analysis

To evaluate the versatility of virus@microbots across different real‐world surfaces and biofluids, a variety of biological and environmental samples were used, including milk, leaf, porcine, and chicken skin. Commercial milk was purchased from a local supermarket and used as a protein‐rich medium to support *S. aureus* growth. With a brief modification of the protocol,^[^ ^74^ ^]^ the milk was inoculated with the bacterial culture and incubated at 37 °C for 4 h in 96‐well plates. Subsequently, the milk was treated with virus@microbots under a magnetic field for 2 h. Post‐treatment, the milk was centrifuged at 6000 rpm, and the resulting bacterial pellet was resuspended in PBS and plated on agar plates. Porcine and chicken skin were sourced fresh from a local butcher shop. Upon collection, both tissues were washed thoroughly and treated with 70% ethanol to eliminate surface contaminants. The samples were then rinsed with sterile PBS and cut to fit within 96‐well plates. Biofilms were cultured on these surfaces by adding *S. aureus* inoculum and incubating at 37 °C for 24 h. Further, unbound bacteria were removed with 1× PBS prior to treatment. For plant surface studies, a fresh maple leaf was harvested directly from a healthy outdoor tree. The leaf was rinsed with distilled water, sterilized with 70% ethanol, and allowed to dry before biofilm inoculation. The biofilm was cultured for 24 h under static conditions, followed by PBS washing and treatment. The samples were subject to virus@microbots treatment under a magnetic field, followed by PBS washing and preparation for SEM analysis across all samples, with a crystal violet assay conducted exclusively for porcine skin.

All samples prepared for scanning electron microscopy (SEM) underwent a standardized fixation and drying protocol. After treatment with virus@microbots, each specimen was fixed in 2% glutaraldehyde for 1 h at room temperature. Subsequently, samples were dehydrated in a graded ethanol series (60%, 70%, 80%, 90%, and 100%) for 10 min each. Once dehydrated, samples were air‐dried, mounted on SEM stubs using carbon tape, and sputter‐coated with a 10 nm gold layer to enhance conductivity and prevent charging during imaging. This method enabled high‐quality visualization of biofilm disruption across diverse biological surfaces using SEM.

## Conflict of Interest

The authors declare no conflict of interest

## Author Contributions

J. performed the conceptualization, synthesis, characterization (SEM, XRD, XPS, and FTIR), data curation, formal analysis, investigation, and prepared figures, compiled and wrote the original draft, and reviewed and edited the final manuscript. S.A. conceptualized microbial studies, conducted SEM analysis, assisted withFTIR, prepared figures, performed data curation, formal analysis, and investigation, and reviewed the draft. X.P. contributed to the synthesis of particles. M.P. did the conceptualization, formal analysis, acquired funding and resources, administered the project, supervised, and reviewed and edited the final manuscript.

## Supporting information



Supporting Information

Supplementary Video1

Supplementary Video2

Supplementary Video3

## Data Availability

The data that support the findings of this study are available from the corresponding author upon reasonable request.
