# Supplementary material for: Virus Enhanced Microrobots for Biofilm Eradication
Source: Adv Mater. 2025 Oct 4;38(2):e08299. doi: 10.1002/adma.202508299 (PMC12783983; doi:10.1002/adma.202508299)
Supplement: Supplementary file 1 — Supporting Information [file ADMA-38-e08299-s002.pdf]

ADVANCED MATERIALS

Supporting Information

for *Adv. Mater.*, DOI 10.1002/adma.202508299

Virus Enhanced Microrobots for Biofilm Eradication

*Jyoti, Sagar Arya, Xia Peng and Martin Pumera**

Virus Enhanced Microrobots for Biofilm Eradication

Jyoti,[1] Sagar Arya,[1] Xia Peng,[1] Martin Pumera[1,2,3,4*]

[1] Future Energy and Innovation Laboratory, Central European Institute of Technology, Brno University of Technology (CEITEC-BUT), 61200 Brno, Czech Republic

[2] Faculty of Electrical Engineering and Computer Science, VSB - Technical University of Ostrava, 17. listopadu 2172/15, 70800 Ostrava, Czech Republic

[3] Department of Chemical and Biomolecular Engineering, Yonsei University, 50 Yonsei-ro, Seodaemun-gu, Seoul 03722, South Korea

[4] Department of Medical Research, China Medical University Hospital, China Medical University, No. 91 Hsueh-Shih Road, Taichung 4040

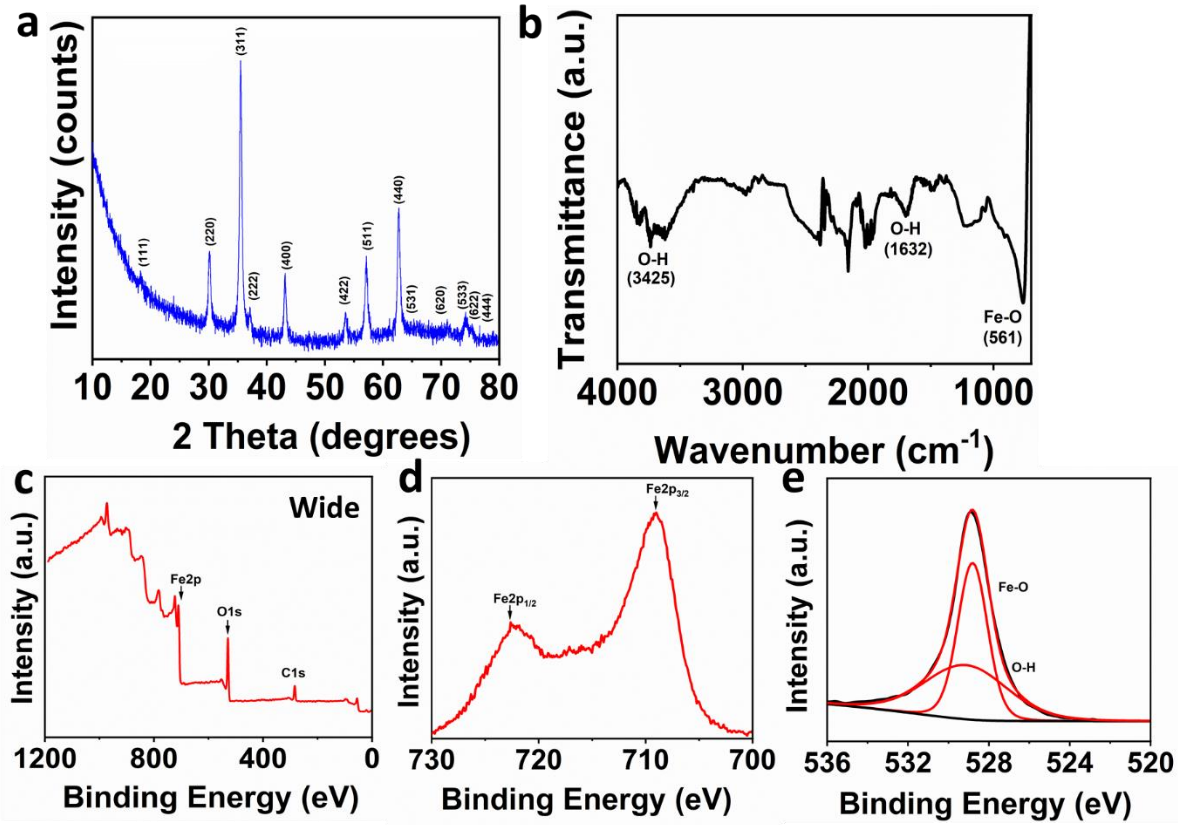

Figure S1: Material characterization of Fe_3O_4 microrobots. a) X-ray diffraction (XRD) pattern showing peaks corresponding to the cubic structure of Fe_3O_4 in agreement with JCPDS Card 75-0033. b) Fourier-transform infrared spectroscopy (FTIR) spectrum confirming the Fe-O vibration and hydroxyl (-OH) groups on the surface of the microrobots. c) Wide-scan XPS spectrum identifying Fe, O, and C as major components of Fe_3O_4. d) Deconvoluted XPS spectrum of Fe 2p, showing peaks for Fe $2p_{3/2}$ and Fe $2p_{1/2}$, respectively, consistent with Fe_3O_4 species. e) Deconvoluted XPS spectrum of O 1s, indicating the presence of metal-oxide and hydroxide bonds.

PART 2: Virus Cultivation and Extraction

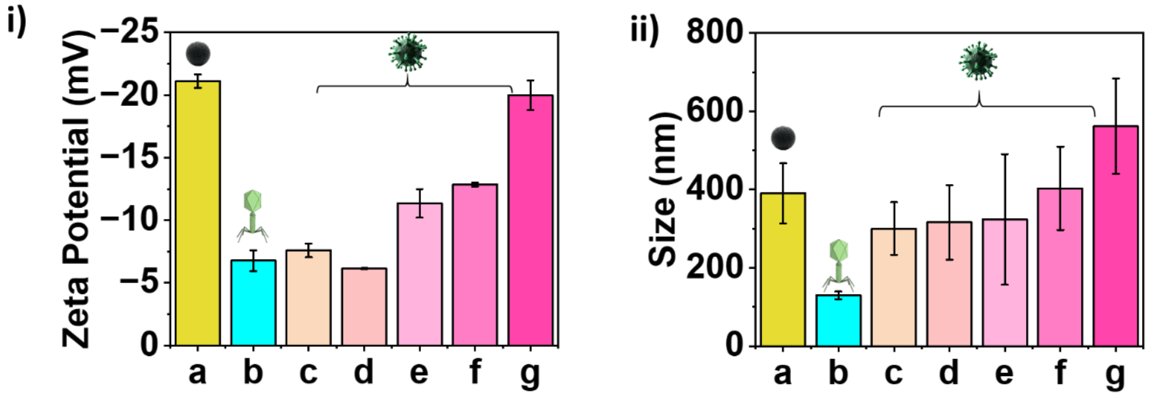

Figure S2: Surface charge and hydrodynamic size analysis of virus@microbots. i) Zeta potential measurements showing the surface charge of bare microrobots, viruses, and virus@microbots by varying the concentrations of the viruses only. ii) DLS analysis illustrating the hydrodynamic size distribution of microrobots, viruses, and virus@microbots (at different concentrations of the viruses). (Condition: Microrobots conc. 0.1 mg mL^{-1}, a) MRs only, b) Viruses only, Virus@microbots ranging from c)10^4 pfu mL^{-1}, d) 10^6 pfu mL^{-1}, e)10^8 pfu mL^{-1}, f)10^9 pfu mL^{-1}, g)10^{10} pfu mL^{-1}

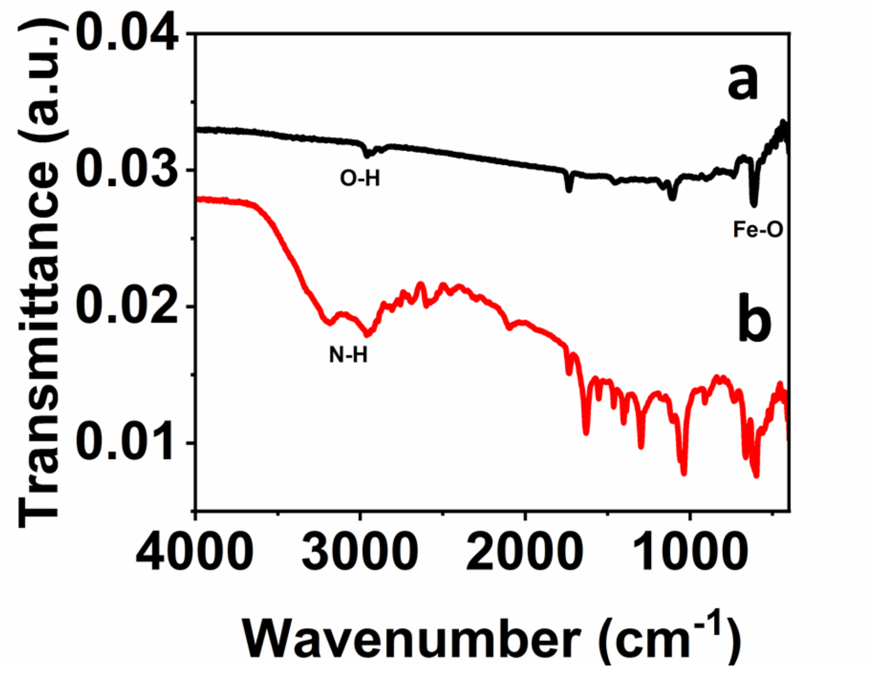

Figure S3: FTIR spectra confirming virus conjugation on Fe_3O_4 microrobots. Spectrum (a) shows characteristic Fe-O stretching (≈580 cm^{-1}) and surface hydroxyl (-OH) groups (≈3000 cm^{-1}) of Fe_3O_4 microrobots. Spectrum (b) displays additional N-H (≈3000-3200 cm^{-1}) and C=O (≈1500-1700 cm^{-1}) peaks, confirming successful viruses' conjugation while retaining the Fe_3O_4 core structure.

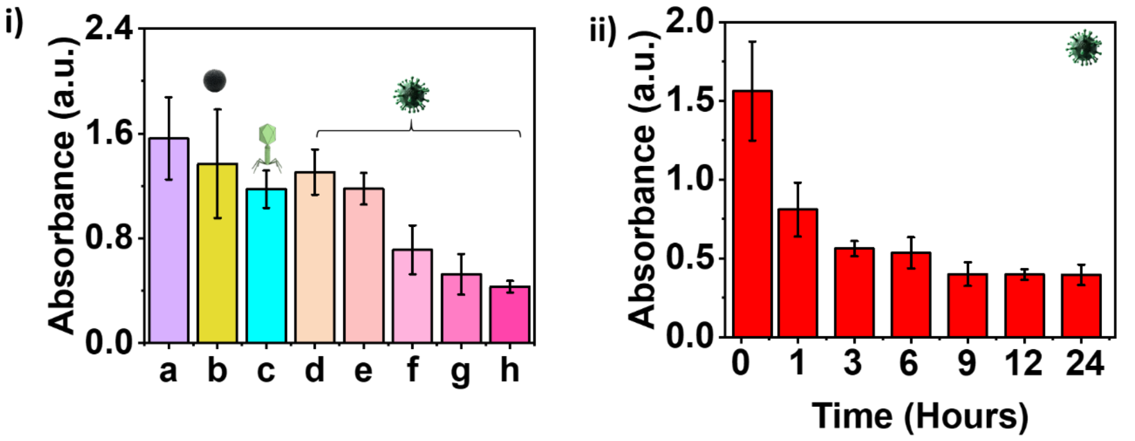

Figure S4: Viruses concentration and time optimization for effective biofilm eradication by virus@microbots. i) Biofilm removal was evaluated at various virus's concentrations ranging from 10^4, 10^6, 10^8, 10^9, 10^{10} pfu mL^{-1}. a) blank control b) microrobots only (0.1mg mL^{-1}) c) viruses only d-h) virus@microbots ii) Biofilm removal was assessed over incubation times of 0–24 hours using optimized conditions (microrobots: 0.1 mg mL^{-1}, viruses: 10^8 pfu mL^{-1}). Error bars indicate standard deviations from three independent measurements.

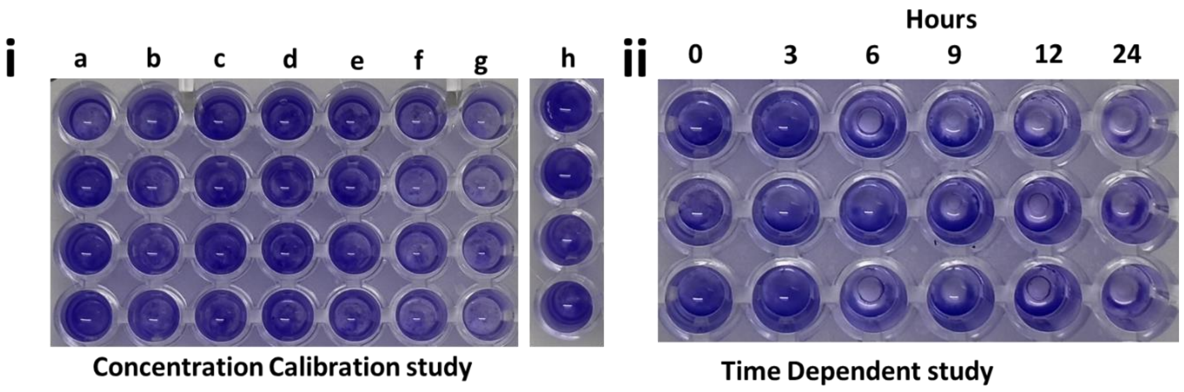

Figure S5: Crystal violet images of the microplate i) showing concentration calibration study of the virus@microbots concentration a) blank b) viruses only c-g) varying virus@microbots concentrations (10^4–10^{10} pfu mL^{-1}) h) MRs only ii) depicting the time dependent study of the virus@microbots (Viruses concentration: 10^8 pfu mL^{-1}; microrobots concentration 0.1 mg mL^{-1}, biofilm grown for 24 hours. Crystal violet 0.1%.). Diameter of well, 6.2 mm.

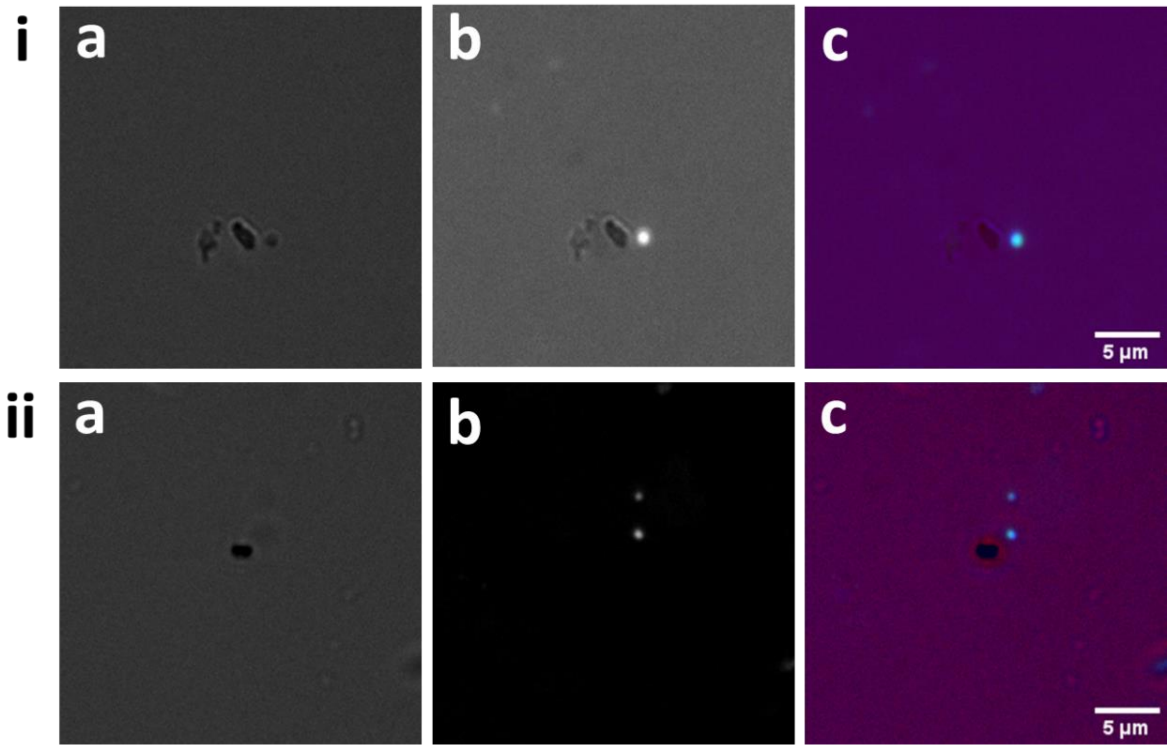

Figure S6: Fluorescence microscopy of bacterial trapping by virus@microbots. Microscopic images of bacterial capture by virus@microbots: i-ii) a) Brightfield mode; b) Fluorescent mode; c) Multichannel mode, showing simultaneous capture and visualization.

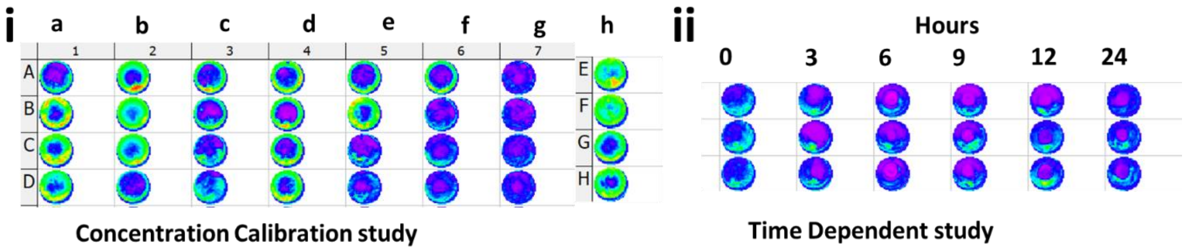

Figure S7: Biofilm mapping for optimization of virus concentration and incubation time. i) illustrates the concentration optimization of virus@microbots with fixed microrobot concentration (0.1 mg mL^{-1}) and a) blank b) viruses only c-g) varying virus@microbots concentrations (10^4–10^{10} pfu mL^{-1}) (h) MRs only. ii) time optimization with fixed virus@microbots concentrations (microrobots: 0.1 mg mL^{-1}, viruses: 10^8 pfu mL^{-1}) across incubation times (0–24 hours). Diameter of the well, 6.2 mm.

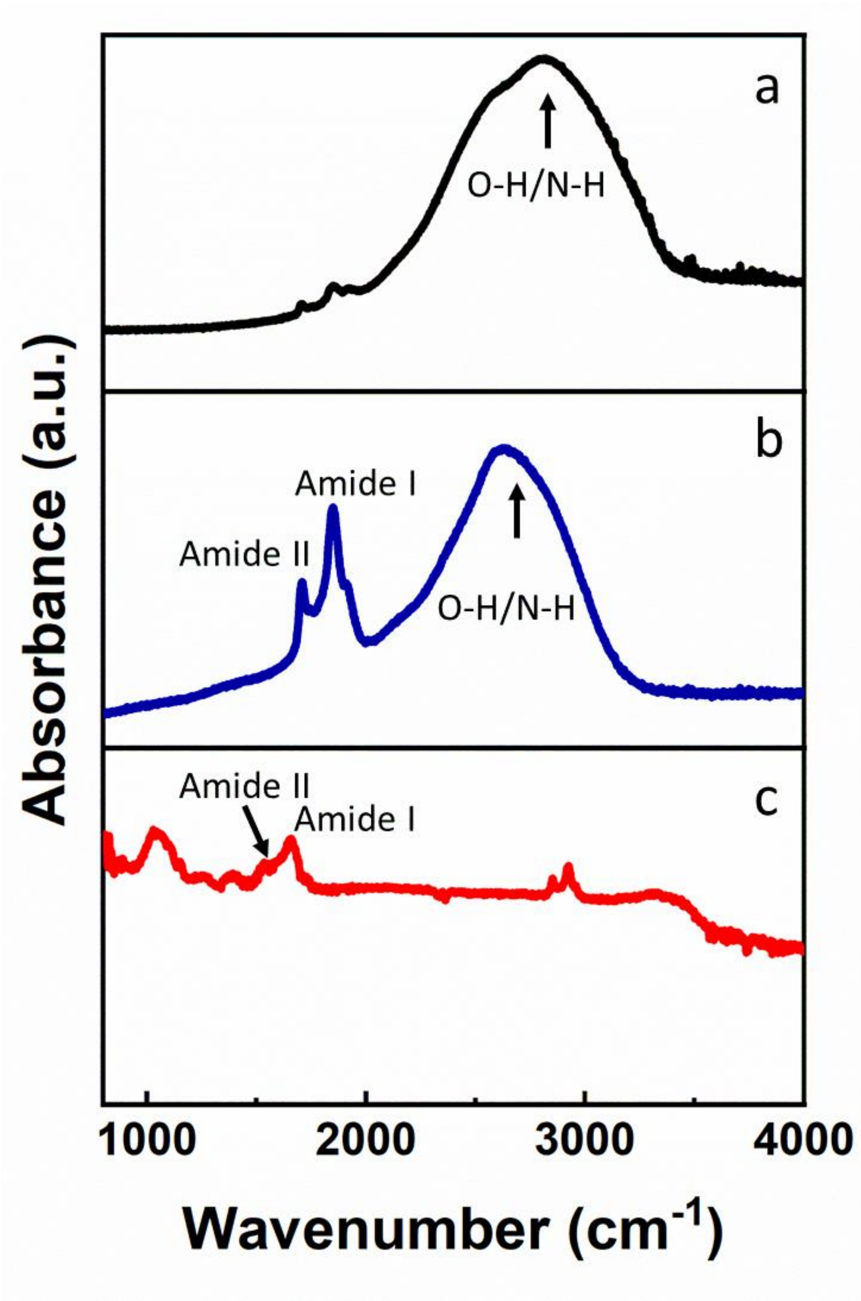

Figure S8: FTIR spectra of a) milk only, displays a broad band around 3300–3400 cm^{-1}, attributed to hydrogen bond stretching vibrations from –OH and –NH groups commonly present in milk. Spectrum b) virus@microrobots in milk, implies that bands become more structured, and additional peaks emerge around ≈1650 cm^{-1} and ≈1550 cm^{-1}, corresponding to Amide I and Amide II vibrations, confirming the presence of phage proteins on the microrobot surface. The appearance of peaks around ≈1050 cm^{-1} also suggests C–O or P=O stretching, indicating surface bioconjugation. In spectrum c), representing virus@microrobots in water, weak absorbance and less defined features reflect the absence of milk matrix, though subtle Fe–O or amide contributions may be present. Collectively, these spectral features confirm successful phage conjugation and the distinct fingerprint of virus@microrobots in biological and aqueous media.
